# Asian elephant calf physiology and mahout perspectives during taming in Myanmar

**DOI:** 10.1098/rsos.231172

**Published:** 2024-04-10

**Authors:** Jennie A. H. Crawley, Hnin Nandar, Htet T. Zaw, Mirkka Lahdenperä, Diogo J. Franco dos Santos, Martin W. Seltmann, Janine L. Brown, Robert M. Goodsell, Zaw M. Oo, Win Htut, U. K. Nyein, Htoo H. Aung, Virpi Lummaa

**Affiliations:** ^1^ Department of Biology, University of Turku, Turku 20014, Finland; ^2^ Myanma Timber Enterprise, Yangon 11011, Myanmar; ^3^ Xishuangbanna Tropical Botanical Garden Institute, Chinese Academy of Sciences, , Yunnan 650023, People's Republic of China; ^4^ Department of Public Health, University of Turku and Turku University Hospital, Turku 20521, Finland; ^5^ Centre for Population Health Research, University of Turku and Turku University Hospital, Turku 20521, Finland; ^6^ Center for Species Survival, Smithsonian Conservation Biology Institute, Front Royal, VA 22630, USA; ^7^ Ecology and Evolutionary Biology, School of Biosciences, University of Sheffield, Sheffield S10 2TN, UK; ^8^ Naturhistoriska Riksmuseet, Stockholm 40 11418, Sweden

**Keywords:** training methods, taming, welfare, physiology, stress, handler

## Abstract

A quarter of Asian elephants are captive, with greater than 90% of these tamed and cared for by handlers (mahouts) in Asia. Although taming is a much-discussed welfare issue, no studies to our knowledge have empirically assessed its impact on calves, and dialogue surrounding taming often lacks perspectives of those involved. Here, we interviewed mahouts involved in taming and monitored five physiological measures (faecal glucocorticoid metabolites (FGMs), serum cortisol, glucose, creatine kinase (CK) and heterophil:lymphocyte (H:L)) over the first 10 days of taming and following six months in 41 calves undergoing taming and 16 control individuals. These measures assess the duration and intensity of stress during taming. Interviews suggested mahouts had major concerns for their safety when discussing changing taming practices, an important consideration for future management. Calf physiological measures were elevated by 50–70% (FGMs/cortisol/glucose), 135% (H:L) and greater than 500% (CK) over the first few days of taming, indicative of elevated stress, not seen to the same extent in control adults. Some measures stabilized sooner (glucose/cortisol/CK/FGM: 7–10 days) than others (H:L: one–two months), indicating mostly acute stress. Our findings inform the welfare of approximately 15 000 captive elephants around the world. Future studies should compare taming in different populations and consider calf and mahout welfare.

## 1. Introduction

As much as a quarter of the world’s total Asian elephant population (approx. 15 000 elephants [1]) live in captivity in Asia cared for by handlers known as mahouts. Asian elephants have long been culturally and economically important in their range countries. They currently vary from individual elephants owned by mahouts and villages, to thousands of elephants managed centrally by governments [2]. The level of human interaction for captive elephants varies from protected-contact (physical barriers separate elephants and handlers) to free-contact (no physical barriers) management [3]. Although protected contact is increasingly used for captive elephant management in the West, the vast majority of captive elephants in Asia are managed in free contact, often required for their work in, for example, logging, forest patrols, tourism, transport or temple ceremonies [2]. As these elephants are not selectively bred to allow domestication, and can pose a threat to their caretakers [4], they are tamed to allow safe handling within their working context.

Thorough documentation of traditional training techniques used across Asia is scarce, though some details exist (Thailand [5], Nepal [6,7], Myanmar [8] and India [9,10]). Training techniques differ both between and within countries but tend to involve a mixture of negative reinforcement (target behaviour removes unpleasant stimulus), positive punishment (unpleasant stimulus decreases unwanted behaviour) and positive reinforcement (target behaviour rewarded). Training generally takes place when calves are 2–4 years old and there are several common practices such as calf separation from the mother, the use of restraints, well-trained chaperone elephants (*kunki*), repetitive songs and tactile training. ‘Positive training’ relying solely on positive reinforcement or pain-free negative reinforcement (pressure as the unpleasant stimulus) [5] is becoming more widespread, especially in Western institutions. Although it is also being increasingly promoted throughout Asia [11,12], it has generally been used after initial traditional taming [13], and the safety of solely relying on positive training in free-contact environments is debated [5,14].

Many taming practices are often assumed to be stressful and to jeopardize elephant welfare [5]. The taming period has been shown to have historically impacted survival, with our study from one of the largest captivepopulations in Myanmar finding a greater than 50% increase in mortality around the taming age of 4 years [15]. Though the mortality rate was shown to have declined significantly in recent years [15], taming is probably still a period of stress for calves. However, to our knowledge, there have been no empirical studies of how traditional taming impacts the elephants experiencing it. Here, we collected longitudinal blood and faecal samples from 41 elephant calves undergoing taming in the same Burmese population to empirically assess their physiological responses, as well as in 16 kunki elephants in the same taming camps, as a comparison.

Specifically, we monitored five measures involved in an individual’s physiological response to stress: concentrations of faecal glucocorticoid metabolites (FGMs), serum cortisol (SC), creatine kinase (CK) and glucose, as well as the ratio of heterophil:lymphocyte white blood cells (H:L). We also interviewed the mahouts involved in the taming process to explore their views and concerns surrounding taming and predict the feasibility of any future management changes. An acute stress response primes the body to respond to stressors to help an individual to cope with the stressful situation. However, when this stress response is prolonged over a long period, known as chronic stress (long-term activation of the hypothalamus–pituitary–adrenal (HPA) axis), it can negatively impact a range of bodily functions, from motor and sensory performance [16], to growth [17], immune function [18], body condition [19] and ultimately reproduction [20] and survival [21]. We were therefore interested in whether there were changes in these measures indicative of stress over the taming period, and what the duration and intensity of these changes were. We collectedfrequent samples to closely monitor physiological changes and measured a range of indicators for a wide view of the stress response [22,23]. We measured glucocorticoid hormones directly from the blood as an immediate indicator (approx. 15 min) of HPA activation as well as metabolites excreted in the faeces as a cumulative measure built up over a matter of hours with a delay of approximately 20–33 h due to digestion [24]. It can be difficult to predict the influence of chronic stress on glucocorticoids, and the direction of change (increase or decrease in HPA activity) may be less important than the change itself [23]. HPA activity has been found to diminish over time with chronic stress, although this depends on the controllability and predictability of the stressor, with restriction-related stress usually resulting in an increased response [23,25]. Another measure we investigated was blood glucose concentration. Although also influenced by nutrition, glucose can indicate stress intensity, increasing following elevated catecholamines and cortisol in the blood, primarily due to the breakdown of glycogen in the liver [26]. Increased heterophils and decreased lymphocyte white blood cells also indicate physiological stress, and the H:L ratio has been suggested to be a useful indicator of chronic stress [22]. Finally, we quantified physical exertion by measuring CK which increases in the blood with muscle damage [27].

We would expect to see short-term increases in all our measures over the first few days if the calves undergo acute stress, and possibly lasting into the following weeks and months if they experience chronic stress as widely assumed, especially in H:L [25]. Pinpointing the highest peaks in these measures will allow us to inform management to pay particular attention to calves during those most vulnerable times. This study sets the foundations for future comparisons of different taming techniques across populations under varying management.

## 2. Material and methods

### 2.1. Study population

The study elephants were managed by the Myanma Timber Enterprise (MTE) whose population comprised more than 3000 semicaptive elephants. Adult MTE elephants worked 5–8 hours per day, were released into the forest when not working, and were rested from work during the hot season (mid-February–May). Each elephant had a logbook detailing demographic and physiological information and a mahout responsible for their daily care. Mothers were rested from work from mid-late pregnancy until their calf was approximately 1.5 years, when they returned to light work with their calf at heel. Calves suckled at will from their mothers and were minimally handled prior to taming.

### 2.2. Taming procedure

Taming occurred in the cold season (November–December), generally of the calves’ fifth year, for calves at least 157.5 cm, though early taming could be requested for those, for example, competing with younger siblings for milk or for calves/mothers particularly difficult to handle. Calves had one month of intensive training first introducing them to human handling at a designated camp followed by continued training over the coming months and years. Taming camps were in well-shaded, level areas of the forest with reliable surrounding fodder and water. The camps generally tamed four or five calves at once, and each calf had a group of mahouts supervised by experienced head mahouts (*sin-gaung*) responsible for their taming, in addition to a veterinarian and regional chief mahout (*sin-oke*) in the camp for the first month. There were a similar number of *kunki* elephants as calves in the camp to control them and minimize direct calf–mahout contact. Kunkis were kept in the same camp as the calves, and it was considered a period of rest relative to usual working tasks, with plenty of time to forage and minimal work compared with logging (e.g. walking with calves and collecting firewood).

On the first day of taming, calves were mildly sedated (0.04–0.14 mg kg^−1^ xylazine, aiming for relaxed trunk and calm demeanour but never unconsciousness) and their mother was led away by their mahout, sometimes with a kunki elephant. While sedated, calves were restrained by ropes to the legs and shoulders and a breast band around the chest to prevent injury to themselves or the mahouts, which were gradually removed over the following week to 10 days. Calves were taken for daily walks (0.5–3 h) supervised by kunkis, to practise carrying a mahout, for exercise and to bathe and drink from a natural water source. In the camp, calves were provided with regular water and fodder collected from the forest by mahouts (e.g. staples: bamboo leaves, paddy, broom grass; and supplements: rice, tamarind with salt, banana stem).

Taming sessions involved the group of mahouts surrounding the calves and rubbing them all over while singing a song, similar to that described in detail in Lainé [9]. The aims of these sessions, according to the mahouts involved, were to familiarizing the calves with the mahouts’ voices and touch and calm them as well as increasing obedience and asserting dominance (see §3). These sessions took place early morning and late evening when it was coolest and lasted approximately 45 min with approximately 30 min of rest in between, repeated around five times a day. As taming went on and the calves became more familiar with the mahouts, the mahouts would begin to introduce and teach the commands and behaviours required for the elephants’ working life (e.g. to move/raise foreleg, lower head, raise trunk, turn left/right, sit).

### 2.3. Mahout interviews

We conducted interviews with mahouts involved in the taming process to understand their points of view on aspects of taming in 2018 (*n* = 71), 2019 (*n* = 54) and 2020 (*n* = 39). Interviews were based on semi-structured questionnaires of 11 questions (see electronic supplementary material, table S1) conducted verbally in Burmese. This may have led to interviewer-based biases but it was done to aid less-literate mahouts, obtain their consent and provide further explanations, if necessary. The questions covered demographic information (Q.1–4), experience in elephant handling and taming (Q.5–7) and opinions around taming techniques (Q.8–11) using both multiple choice and open-ended questions to obtain definitive answers while also allowing mahouts to explain their choices.

### 2.4. Study animals

We studied 41 calves tamed in two locations in the Sagaing region of Myanmar: 9 in November–December 2018 (3 females and 6 males), 17 in 2019 (7 females and 10 males) and 15 in 2020 (7 males and 8 females); see electronic supplementary material, table S2(i) for an overview of calves. Calves were 3.5–4.9 years old (average 4.4 ± 0.06) at the start of taming and their shoulder height was 154–185 cm (average 170.6 ± 1.71). We started faecal sample collection before taming began to try to establish a baseline FGM concentration. Elephants had often already travelled to taming camps which may have elevated these baselines, though we do not foresee this having a large influence. Journey times could vary from less than half an hour to days; however, those that travelled furthest often arrived days in advance to rest before beginning taming. The journey was always done by foot and would not be an unusual occurrence for calves as they often leave their camps to accompany their mothers, for example, to attend to light working duties. Furthermore, other measures were taken to minimize the stress of the journey, such as allowing plenty of time for foraging and resting along the way and bringing siblings and allomothers along. Blood samples could only be taken once taming had begun, and although we consider month 6 to be a baseline of sorts as most of the measures had visually stabilized by then, patterns should be interpreted with this in mind. We took samples daily from calves for the first 10 days (days 0–9) where possible, followed by monthly collections over six months (see table 1). We also monitored 16 kunki elephants over the first 10 days (1 female and 15 males), as control elephants in the same environment not experiencing taming. The kunkis were 12.4–60.0 years old (average 36.5 ± 3.9, see electronic supplementary material, table S2(ii)). We took daily faecal samples from kunki and blood samples at days 0 and 9, but kunki blood sampling on days 1–8 was limited (table 1) and we were unable to monitor kunkis in the following months. All data were collected by MTE veterinarians under the approval of the MTE, collected as part of the elephants’ routine monitoring and were deemed not to require a project license according to the Finnish National legislation (Act 497/2013 and Decree 564/2013 on the protection of animals used for scientific or educational purposes) or the EU Directive 2010/EU/63 on the protection of animals used for scientific purposes.

**Table 1.** Sample collection overview showing number of samples collected for each data type each day for both calves and kunkis.

		days since taming started																			months since taming started					
measure	Ele ID	−11	−10	−8	−6	−5	−4	−3	−2	−1	0	1	2	3	4	5	6	7	8	9	1	2	3	4	5	6
FGMs	calf	3	4	5	4	4	5	6	5	32	40	40	40	39	40	39	40	40	40	40	38	38	37	38	36	37
	kunki	—	1	4	—	—	—	3	1	36	43	40	47	50	48	43	37	36	36	30	—	—	—	—	—	—
SC	calf	—	—	—	—	—	—	—	—	—	18	10	9	9	14	15	14	15	14	31	28	28	29	26	27	25
	kunki	—	—	—	—	—	—	—	—	—	10	5	6	—	—	—	—	—	6	11	—	—	—	—	—	—
CK	calf	—	—	—	—	—	—	—	—	—	22	10	10	7	12	10	15	8	8	33	23	22	23	2	23	22
	kunki	—	—	—	—	—	—	—	—	—	9	9	6	—	1	—	—	—	—	21	—	—	—	—	—	—
H:L	calf	—	—	—	—	—	—	—	—	—	33	22	22	29	29	28	29	24	29	38	38	38	38	38	37	37
	kunki	—	—	—	—	—	—	—	—	—	22	8	7	—	7	4	—	—	—	33	—	—	—	—	—	—
glucose	calf	—	—	—	—	—	—	—	—	—	33	24	28	35	35	38	38	37	38	37	38	38	38	38	37	37
	kunki	—	—	—	—	—	—	—	—	—	22	9	7	—	7	4	—	—	—	33	—	—	—	—	—	—

### 2.5. Sample collection

#### 2.5.1. Faecal samples

The faecal sampling protocol is shown in table 1. Samples collected 1–11 days prior to the start of taming varied due to arrival times at the camp depending on travelling distances from pre-taming locations. Faecal samples were always collected in the morning to reduce diurnal variation and frozen until drying for shipment and analysis at the Chiang Mai Veterinary Diagnostics laboratory, Thailand. FGM concentrations were measured using an enzyme immunoassay based on a polyclonal rabbit anti-corticosterone antibody [28], used previously to assess stress status in elephants [29]. The intra-assay variation was less than 10%, inter-assay variation was 12.34% and the detection limit was 0.1212 ng ml^−1^.

#### 2.5.2. Blood samples

Blood sampling frequency is also shown in table 1. MTE veterinarians took blood early in the morning from an ear vein into vacuettes containing either ethylenediaminetetraacetic acid (H:L) or serum separator/clot activator (cortisol/CK). Glucose was measured immediately from a drop of blood using an ACCU-Chek Aviva glucometer. To calculate H:L, we stained blood smears with Romanowsky stain and used an optical microscope (40× magnification) to visually classify 100 cells as monocytes, heterophils, lymphocytes, eosinophils or basophils from which we calculated the H:L ratio (as in [30]). Serum separator tubes were centrifuged for 20 min at 3400 r.p.m. to obtain serum, which was then frozen (at −20/−80°C) until analysis. Sera were analysed for CK at the Crown laboratory, Yangon, Myanmar using an IDEXX VetTest® analyser (minimum detection 9 U l^−1^). Sera were analysed for cortisol at the University of Turku, Finland, using a multi-species enzyme immunoassay (detailed protocol in electronic supplementary material, Supplementary Information). For this assay, we carried out serial dilutions to determine the optimal dilution of serum samples to be 1:32 at which the majority of samples were analysed, unless they fell outside the curve in which case we reanalysed at 1:64. The intra-assay coefficient of variation was less than 10%, inter-assay variation was 17.4%, and the limit of detection was 34.69 pg ml^−1^.

### 2.6. Statistical analysis

#### 2.6.1. Models a–e

We fit generalized additive models using the *mgcv* package [31] in R (v. 4.2.2 [32]) to the calf physiological data: (i) daily over the first 10 days; and (ii) monthly over the following six months, to assess temporal trends. Models included either (a) FGMs, (b) cortisol, (c) glucose, (d) H:L or (e) CK as the response variable, with Gaussian distributions (a), (d) and (e) log-transformed using the natural logarithm (ln)). We included a penalized thin plate regression spline for the term of interest, either (i) taming day (18 knots in model a; 10 knots in models b–e) or (ii) taming month (six knots), a fixed effect for location (binary: 1/2), calf age at the start of taming (mean-centred and scaled), the year (2–3 levels factor: 2018/2019[/2020]) and sex (binary: male/female) and a random intercept spline of elephant ID (factor: 19–35 levels). We tested whether including an interaction between the time and sex variables improved model fit in case the temporal pattern differed between males and females. We determined these were important variables to include as taming treatment or environment may differ between camp locations and years, and taming experience and ability to cope may differ between calves of different ages and sex. We also assessed temporal patterns of kunki FGMs over the first 10 days (model aKi) as kunki faecal samples were also collected daily, using a separate model as kunkis differed more in variables such as age than the calves. The terms were generally similar in the kunki model, though we included age at measurement (mean-centred and scaled) rather than age at the start of taming. We included a term for days between serum collection and analysis in the CK models to account for known storage time effects (73–1059 days, mean = 353 ± 9.95).

We present model outputs in the electronic supplementary material, Supplementary Information, and give relative Akaike information criterion (AIC) values of models including and excluding terms of interest, used to interpret model fit, in the results. In the years 2019 and 2020, taming was staggered within the same camps, and therefore the date of the first day of taming varied for different calves within the same location. Therefore, kunki samples from the same day could be classed as ‘day 0’ and ‘day 2’ simultaneously, and therefore were included twice, hence why there can be more samples recorded some days than kunkis in table 1.

#### 2.6.2. Estimating peak values

We estimated the time of the maximum value for each response variable, referred to as the ‘peak’, by simulating values from the posterior densities of the parameters of the fitted models. This involved repeated prediction of the response variable for simulated combinations of coefficients (marginalized over other variables) to find the optima of the simulated curves. From the resulting distribution of maximal values, we calculated the mean and 95% confidence intervals to represent the estimated peak of the curve and uncertainty. Again, we only calculated a ‘kunki peak’ for the FGM measures taken in the days prior to and in the first 10 days of taming.

## 3. Results

### 3.1. Mahout perspectives on taming approaches

Our interviews with the mahouts explored their views on taming and their attitudes towards different approaches and making any changes to current procedures. An overview of the interview questions and their response type (e.g. open ended/multiple choice) are given in electronic supplementary material, table S1. Each calf had two–four head mahouts supervising their taming, four–seven mahouts helping and one assigned future mahout. The future mahouts had 3.7 ± 1.2 years of experience working with elephants on average, and only 36% had previously been involved in taming. The helping mahouts had 4.0 ± 0.6 years of experience and 79% had experienced taming before. Head mahouts had 12.4 ± 1.0 years of experience and 84% had experienced taming, having been involved up to 17 times, on average 5. Head mahouts were 24–48 years old (average 36 ± 1.0 years), future mahouts 15–29 (average 20 ± 1.2) and the helping mahouts 11–42 (average 23 ± 0.7). When asked about the function of the singing and rubbing process (Q.8, respondents *n* = 120), there was a mixture of answers, which we grouped into general categories, with 52% mentioning it helps make the calves ‘clever’ (generally used to denote obedience rather than intelligence), 29% as a way to ‘dominate’ the calves, 19% to ‘familiar[ize]’ the calves to mahouts’ voices and touch or improve trust between them, 8% to help the calves to focus or learn (some mentioned more than one purpose) and 4% to reduce fear.

When asked about their emotions when the calves were in the breast band (Q.9, respondents *n* = 163), 63% felt ‘pity, but it is necessary’, 21% of respondents said they felt ‘sad’ and 16% felt ‘normal’. When asked about their feelings on positive reinforcement training (Q.10a/b, respondents *n* = 164/145), phrased as ‘do you think it would be possible to tame only with reward and no punishment?”, 60% answered ‘no’, 26% ‘yes’ and 12% ‘sometimes’. Of those who answered ‘no’, many were worried it would pose a danger to mahouts (68%), either immediately with a threat of crushing (53%) or later with mahouts unable to trust their elephants (12%). Of those who answered ‘sometimes’, many expanded that although it might be possible, the elephants (trained using this method) could not necessarily be trusted (67%), especially in the context of working elephants. Of those 25% who answered ‘yes’, 41% thought softer methods would give better results, while one-third still thought traditional taming is better or safer (31%). Many also mentioned soft training taking more time (22%) as both a limiting factor, but also leading to better results. When asked about any problems in the taming procedure and whether they would like to make any changes (Q.11a/b, respondents = 155/156), most said they were not experiencing problems and had no suggestions (74%), 18% suggested changes, 9% thought it was better recently and 8% mentioned bad calf behaviour. Suggested changes included reducing punishment/restraints for the calves, fasting the calves as is routine for private elephants, improving training group relations and providing more food/blankets for the mahouts.

#### 3.1.1. Faecal glucocorticoid metabolites

**Figure 1.**
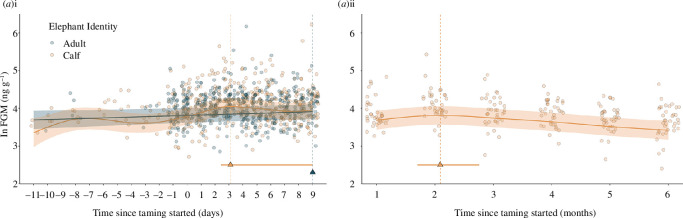
Faecal glucocorticoid measures *a*) during the (i) 11 days leading up to and the 10 days during, and (ii) the 6 months following, the most intensive taming period. Circular points show the raw data and filled lines with associated shaded areas show model predicted values and 95% confidence intervals, with marginal predicted values for an average-age female calf/male kunki from location 1 in 2018. Triangular points and associated lines show maximum values and 95% confidence intervals around this peak calculated via posterior simulations for female calves/male kunkis in location 1 in 2018. Colours show elephant identity (orange: calf, grey: kunki).

Calf FGM concentrations ranged from 11.1 to 506.8 ng g^−1^ (mean ± s.e.: 61.5 ± 1.39), while kunkis in the same environmental conditions ranged from 17.4 to 479.8 ng g^−1^ (62.1 ± 1.54). The estimated peak of calf FGMs was on day 3, but confidence intervals spanned days 3–9 (max. = 3.1, lower CI = 2.4, upper CI = 9.0). The estimated peak for kunki was at day 9 (max. = 9.0, lower CI = 9.0, upper CI = 9.0). Calf changes in FGM from before taming (days −11 to −1 combined) to the peak at day 3 ranged from −17% to 492%, with a median increase of 51%, whereas kunki changes ranged from −33% to 154% in the same time, with a median 17% increase. Figure 1*a*(i) shows calf FGM concentrations were visually more variable and higher than kunki concentrations between days 2 and 5, though the confidence intervals overlap. The time variable improved model fit in both models: *a*(i) (ΔAIC −52.34) and *a*(ii) (ΔAIC −32.14), suggesting the changes in calf FGMs seen over both the first 10 days and the following six months were significant (differed from a flat horizontal line), as was also the case for the kunkis over the first 10 days *a*(i) (ΔAIC −2.73), though not as strongly (see electronic supplementary material, table S3). The interaction between sex and time improved model fit in *a(*i) (ΔAIC −8.92) but not in *a*(ii) (ΔAIC +7.74), suggesting that there were sex-specific differences in calf FGMs change over the first 10 days but not in the following months (electronic supplementary material, table S3). As seen in electronic supplementary material, figure S1*a*(i), the FGM concentrations of females were visually slightly more changeable than male concentrations over the days leading up to and the first 10 days of taming, but confidence intervals overlapped for most of the time. As shown in figure 1*a*(ii), calf FGM values over the following months were highest between 2 and 3 months after the start of taming (max. = 2.1, lower CI = 1.7, upper CI = 2.8). Although FGM levels declined towards the end of the six-month period, they appear to reach similar values to the baseline samples collected prior to taming rather than suggesting chronic stress-induced hypocortisolism.

#### 3.1.2. Serum cortisol

**Figure 2.**
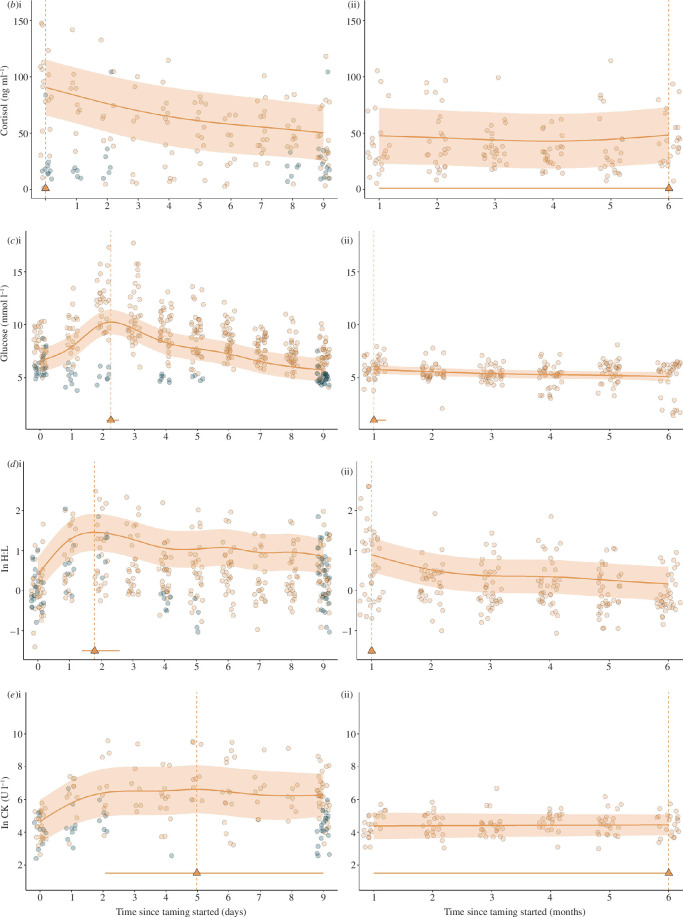
Physiological stress markers (*b*) serum cortisol, (*c*) glucose, (*d*) ln heterophil:lymphocyte and (*e*) ln creatine kinase over (i) the first 10 days of taming and (ii) the following 6 months. Circular points show the raw data and filled lines with associated shaded areas show model predicted values and 95% confidence intervals, with marginal predicted values of female calves in location 1, and male kunkis in location 1. Triangular points and associated lines show maximum values and 95% confidence intervals around this peak calculated via posterior simulations for males in location 2. Colours show elephant identity (orange: calf/ black: kunki).

SC concentrations ranged from 2.6 to 147.7 ng ml^−1^ in calves and 1.4–104.4 ng ml^−1^ in kunkis, with the average cortisol concentration of calves higher than kunkis at 47.2 ± 1.8 ng ml^−1^ and 24.4 ± 3.4 ng ml^−1^, respectively. The taming day term improved model fit in model *b*(i) (ΔAIC −21.15; electronic supplementary material, table S4) suggesting the changes in calf cortisol over the first 10 days were significant with cortisol at its highest concentration on the first day of taming, when the peak was estimated (max. = 0.0, lower CI = 0.0, upper CI = 0.0; see figure 2*b*(i)). Changes in calf cortisol concentrations between days 0 and 9 varied from −96% to 161%, with a median 67% decrease, whereas kunki cortisol remained relatively stable, varying from −76% to 2387% in the same time frame, but with a median 18% decrease. Calf cortisol, however, was stable over the following six months; the taming month term in model *b*(ii) did not improve model fit (ΔAIC +2.57) and the confidence intervals of the simulated peak spanned months 1–6 (max. = 6.0, lower CI = 1.0, upper CI = 6.0: see figure 2*b*(ii); electronic supplementary material, table S4), with no evidence of chronic stress-induced changes to HPA activity. The interactive sex term did not improve the model fit of *b*(i) (ΔAIC +0.50) in the first 10 days (see electronic supplementary material, figure S2*b*(i) or in model *b*(ii) (ΔAIC +0.72; electronic supplementary material, table S4) over the following months, suggesting patterns were similar in males and females (electronic supplementary material, figure S2*b*(ii).

#### 3.1.3. Glucose

Glucose concentrations were higher and more variable in the calves than the control kunkis, ranging from 4.1 to 17.7 mmol l^−1^ (average 8.9 ± 0.09) and 3.8–8.7 mmol l^−1^ (average 5.4 ± 0.07), respectively. The peak for the calves was estimated at day 2 (max. = 2.3, lower CI = 2.1, upper CI = 2.5, figure 2*c*(i)), with changes in this time ranging from −4% to 142%, with a median 49% increase from days 0 to 2, decreasing again by day 9 by a median of 41%. Although blood was not collected daily from all kunkis, the glucose concentrations of those we collected from remained relatively stable over the same time frame, with changes ranging from −31% to −7%, with a median 22% decrease from days 0 to 2 and decreasing again by 3% by day 9 (see figure 2*c*(i)). Calf glucose concentrations remained stable over the following 6 months, though they were highest in month 1 (max. = 1.0, lower CI = 1.0, upper CI = 1.2, figure 2*c*(ii)). The time variables improved the model fit of both models *c*(i) (ΔAIC −190.80) and *c*(ii) (ΔAIC −7.75), suggesting the differences we saw over time were significant both in the first 10 days and the following six months. The interaction between the sex and time variables improved model fit in *c*(i) (ΔAIC − 8.92) and *c*(ii) (ΔAIC − 3.08), suggesting that changes in glucose over time were sex specific in both the first 10 days and the following months (see electronic supplementary material, table S5). As shown in electronic supplementary material, figure S2*c*(i), females had lower values than males generally, except for day 2 where the pattern differed more from a flat line, reflected in their higher effective degrees of freedom (*edf)* value (see electronic supplementary material, table S5), yet there was no observational evidence of sex-specific differences in the following months (electronic supplementary material, figure S2*c*(ii).

#### 3.1.4. Heterophil:lymphocyte

The H:L of calves ranged from 0.24 to 13.5 (average 1.87 ± 0.08) compared with 0.36–7.75 (average 1.73 ± 0.16) for the kunkis. The time variable improved model fit in models *d*(i) (ΔAIC −88.46) and *d*(ii) (ΔAIC −42.24), suggesting the differences seen over time were significant in both the first 10 days and the following 6 months. The peak calf H:L ratio was estimated at day 2 (max. = 1.8, lower CI = 1.4, upper CI = 2.6; figure 2*d*(i); electronic supplementary material, table S6). The change in H:L from days 0 to 2 varied from −31% to 1100%, with a median 135% increase, decreasing again by a median of 40% on day 9. Although not collected daily from every kunki, the H:L ratio for the 7 kunki we collected from showed a similar pattern to the calves, with a median increase of 155% from days 0 to 2 (range 93–263%), decreasing again by 37% on day 9. H:L ratio still appeared to be elevated in the calves 1 month after taming, with month 1 shown as the peak compared with other months (max. = 1.0, lower CI = 1.0, upper CI = 1.0), and seemed to stabilize after only two months (figure 2*d*(ii)). The interactive sex model did not show improved model fit (electronic supplementary material, table S6; figures S2*d*(i and ii) for model *d*(i) (ΔAIC +6.86) or *d*(ii) (ΔAIC +5.83), suggesting patterns over time were not sex specific in either the first 10 days or the following months.

#### 3.1.5. Creatine kinase

CK concentrations were more variable in calves (range: 14–14 462 U l^−1^), and substantially higher on average (829.1 ± 136.9), than kunki CK levels (114.1 ± 19.80), which ranged from 11 to 783 U l^−1^. The time variable improved model fit in model *e*(i) (ΔAIC – 21.94), but not model *e*(ii) (ΔAIC + 1.92; electronic supplementary material, table S7), suggesting that the temporal variation over the first 10 days was significant but not in the following months. The peak of CK in the first 10 days was estimated at day 5, but concentrations increased steeply after day 0 and remained elevated, with confidence intervals of the peak spanning days 2−9 (max. = 5.0, lower CI = 2.1, upper CI = 9.0; figure 2*e*(i)). Changes in calf CK concentration varied from −18% to 10 600%, with a median increase of 591% between day 0 and day 9 but levels differed substantially between individuals, evidenced in the large s.e.s. CK concentrations of kunkis in the same time frame increased to a lesser extent, with changes ranging from −30% to 63% with a median of 35% increase, though we only sampled eight kunkis on these days for CK. The confidence intervals of the peak for calves in the following months spanned 1–6 (max. = 6.0, lower CI = 1.0, upper CI = 6.0), suggesting CK values were relatively stable over this time. The changes in calf CK were similar for males and females, and the inclusion of an interactive sex term did not improve model fit in model *e*(i) (ΔAIC +9.87) or model *e*(ii) (ΔAIC +7.33) (electronic supplementary material, figure S*e*(i and ii); table S7).

## 4. Discussion

The traditional elephant taming procedure has received international attention and criticism for the impacts it has on calf welfare, yet these impacts have never been empirically assessed. Here, for the first time, we collect data during this process to monitor the response of five physiological measures to the traditional taming procedure used routinely for training in the largest population of captive Asian elephants. We monitored calves both in the critical first 10 days of taming and the following six months, to assess the extent and duration of any effects. All five physiological measures increased substantially within the first few days, which could suggest the calves were experiencing an acute stress response. Measures were elevated by 50–70% (FGMs, SC, glucose), 135% (H:L) and greater than 500% (CK) in calves undergoing taming, which were mostly higher than in kunkis. Most measures increased 2–3 days into taming, though cortisol was elevated from the first day. Whereas CK, FGM and cortisol seemed to stabilize after the first week, H:L and glucose appeared to take one–two months to fully stabilize, though this was not as marked in glucose. Although neither of our glucocorticoid measures showed evidence of long-term dysregulation of the HPA axis often associated with chronic stress, elevated H:L lasting up to two months may suggest a chronic stress response. Male and female calves differed in terms of their FGMs over the first 10 days and glucose levels over the whole time period, but there were no strong sex-specific trends.

Although all five physiological indicators were elevated during the first 10 days of taming, the severity, timing and duration of increases differed between measures and individuals. Cortisol was elevated from the first day of taming and steadily declined, while glucose and H:L peaked after two days and FGMs and CK rose after two to three days and remained elevated until day nine. The peaks in glucose and H:L on day two probably reflect a delayed response to the stress of the initial separation from the mother and rope restriction on the first day of taming, whereas these effects were reflected immediately in the SC, a more responsive measure [24]. The peak in cortisol on the first day could also be reflecting the stress of having blood taken for the first time, as SC is a highly responsive measure. However, as calves were sedated during blood-taking on the first day and are unlikely to become accustomed to it in just one day, this is unlikely. Although the highest peaks lasted only approximately a day for glucose, cortisol and H:L, both FGMs and CK remained elevated for over a week and H:L only appeared to fully stabilize after two months. This was reflected in the highest H:L measure in the six months following taming being estimated at month one, suggesting that there may be chronic stress in the first or second month or two. Similarly, the glucose measure in the six months following taming was also highest at month 1, though it had already decreased substantially from the initial peak. In the months following taming, FGM concentrations were elevated after two–three months, but this is probably reflecting seasonal differences as discussed in [33]. A study on laboratory rats showed that while elevated serum corticosterone indicated acute stress, chronically stressed rats showed elevated H:L but not corticosterone concentration [22]. Adrenal hormones are more dependent on an individual’s nutritional or physiological status [22], and may be more prone to habituation (a decline in response to the same stressor over time [34]), or adrenal exhaustion (breakdown of a physiological system leaving an animal too fatigued to maintain a response [35]), which could explain why we see disparities between the duration of H:L effects compared with the other measures. This could be tested for in future studies by injecting corticotropin-releasing hormone or adrenocorticotropin to assess adrenal responsiveness and pituitary function, respectively (ACTH/CRH challenges) [25]. Finally, there were several non-focal variables which significantly influenced the physiological variables, such as elephant ID, taming year and location. Although these are beyond the scope of this study, it would be interesting for future studies to focus on factors driving these differences.

The elevated CK values seen over the first 10 days probably reflect muscle damage and exertion caused by the calves struggling against their rope restriction, which stabilized in the following months when the ropes were no longer present. Similar increases in CK have been reported in response to physical stress during transportation of livestock and wild ungulates, along with increased cortisol, H:L and glucose [26,36–39]. Although we saw some extreme increases in CK, there were a many variations between individual calves (varying from an 18% decrease to a 10 600% increase between days 0 and 9). In fact, four out of five variables showed greater variability between individual calves than between kunkis, and it would be interesting for future studies to investigate whether certain traits or taming/management strategies are linked to calf-specific differences in this and other measures. Further understanding of individual variation could even factor into management decisions regarding selecting particular individuals for, for example, training/breeding/reintroduction based on their response, which is becoming increasingly relevant in elephant conservation discourse. Generally, identifying the greatest period of acute stress as the first few days of taming should inform management decisions to focus care on this critical period, as well as providing concrete empirical evidence of a physiological stress response to encourage caretakers to develop ways to minimize harm during this time. We also recommend continued close attention throughout the first two months when the calves may be experiencing prolonged stress.

It is important to note that xylazine can increase glucose levels for up to 8 hours, and therefore the glucose levels on the first day may not represent an accurate baseline [40]. However, the values from month 6 probably represent normal levels as they appear to stabilize from months 2 to 6, and the average calf glucose concentration of 5.0 mmol l^−1^ falls within the normal reference intervals of the population (2.5–5.5), compared with 8.0 mmol l^−1^ on day 0. Furthermore, xylazine has also been shown to suppress plasma cortisol [36], so the elevated cortisol values seen on the first day may even underestimate the true increase.

Some of the physiological measures of the kunki elephants also increased over the first few days of taming, with FGMs increasing by 17% from days 0 to 3 (compared with 51% in the calves), H:L increasing by 155% from days 0 to 2 (similar to the 135% in the calves) and CK increasing by 35% from days 0 to 9 (compared with 591% in calves). Unfortunately, we were unable to consistently collect blood samples from kunki daily, so the sample size for some of these comparisons are limited (FGMs, *n* = 30; H:L, *n* = 7; and CK, *n* = 21, see table 1). Future studies should extend assessments to include a greater number of adult elephants in the taming environment, and handlers should consider in the meantime that taming may not only negatively influence the calves undergoing taming, but also adult elephants present within the environment. It is also possible there were environmental conditions independent of taming exacerbating the stress or immune responses of both calves and kunkis, such as hot temperatures, which could also be accounted for in future studies.

As no other studies to our knowledge have collected data from elephant calves during taming, it is hard to draw specific comparisons to other taming or management techniques. One study comparing cortisol concentrations of more than 100 zoo elephants in either free contact or protected contact found no difference between management styles, though data were not collected during training [41]. Studies comparing training techniques in dogs [42,43] and horses [44,45], found individuals trained using aversive techniques to exhibit more stress-related behaviours (e.g. lip licking, yawning and panting [42,44]). Aversively trained dogs also generally showed elevated cortisol levels compared with those trained using positive reinforcement [42,46,47] though not in all studies [43]. These studies focused on short-term effects (within 1 hour), but cognitive bias tests showed that aversively trained dogs were more pessimistic up to a month after training [42]. Another study found no evidence that using shock collars affected dog behaviour after 1 year, when used predictably by skilled trainers [48]. Studies have also monitored the physiological stress response to transportation [37–39,49–52], finding the same physiological measures to increase following transportation, though the study durations were also short. Furthermore, increases were comparable to, or surpassed those in this study. For example, one study of cattle found 818% and 135% increases in CK and H:L, respectively, following 24 hours of transportation [38], concurrent with other studies [49]. Effects appear to be especially pronounced for free-ranging animals, with increases in H:L and CK of approximately 800% and 2900%, respectively, following 100 minutes of transportation in free-ranging chamois [38], and 284% and 616% after 9 hours of transportation in free-ranging mouflon [37]. Similar effects have been found in studies on elephants: elephants showed significantly higher FGMs following translocation, with effects lasting 2 days [52]. Although previous studies of translocation stress mostly focused on short-term physiological changes, it is interesting that the magnitude of some of the changes were comparable to our findings, and one study monitoring translocated elephants over a longer timescale found FGMs to remain elevated for up to one month [51], a similar duration to our findings. Future studies of the physiological stress response to different handling methods would benefit from monitoring individuals over a longer timescale to understand the duration of effects, and studies of training should extend to compare different training activities to put changes into context. A further development would also be to assess the optimum age for socialization and taming in elephant calves and to compare the trade-off between benefits gained from introducing more gradual, softer training at an earlier age against the costs of reducing the time calves spend undisturbed with their mother. The taming age in Myanmar is at the older end of the spectrum compared with other captive elephant populations, in order to prolong the time with their mother and increase their ability to cope with taming (discussed more in Crawley *et al*. [15]).

Male and female calves may receive differential treatment during taming, which could have been reflected in their physiological measures. Males are often seen to pose more of a threat and therefore could be treated more harshly. Though there was little sexual dimorphism in body size in our study calves (female height: 169.3 ± 0.9 cm; male height: 171.4 ± 0.8 cm), 15/23 male calves had substantial tusks about which both mahouts and vets voiced concerns. Although we saw sex-specific trends in glucose and FGMs, these differences were not substantial, with confidence intervals mostly overlapping and not indicating one sex as necessarily more vulnerable. Past studies of this population also showed that although juvenile mortality is generally higher in males [53], mortality at taming ages was not [15]. In fact, traits associated with being smaller or younger were generally linked to higher mortality during taming [15], although age at the start of taming did not explain much variation in our models in the present study. Females are anecdotally considered more vulnerable during taming, with softer taming methods often advised for MTE females, but this was not reflected in their physiological measures, perhaps as softer methods mitigated any differences. Further investigation into individual differences in taming responses would help to identify traits associated with different physiological responses to taming.

Mahout interviews provided their perspectives on taming which should help assess the feasibility of adjusting taming approaches in the future. Over 80% of mahouts felt either sad or pity for the calves, suggesting they care about calf well-being, mirroring past descriptions of Thai trainers as ‘good natured people’ [5]. This may seem an obvious observation, but it is not how they are always portrayed. Only 26% of mahouts considered positive reinforcement training possible, with many citing the danger posed by the calves as a counter-argument, and even those who thought it hypothetically possible worded concerns in the context of working elephants. These fundamental safety issues overshadowed concerns about increased time and money, which would have been simpler barriers to overcome. Mahouts were not opposed to considering alternative training methods generally, with many stating a desire to reduce risks for the calves. Importantly, many voiced great respect for the ways of senior mahouts, deferring to their expertise, and suggesting any adjustments must be done in close collaboration with the senior mahouts. Many responses highlighted the risk to mahout safety both during taming and in the future working with trained elephants. This is crucial, as although we may expect elephants trained using positive training techniques to see welfare benefits, behavioural outcomes are likely to differ. For example, zoo elephants trained in protected contact using positive reinforcement refused commands more and took longer to respond than those in free contact using negative reinforcement [54]. This could have important implications considering the danger elephants can pose to their mahouts in a working, free-contact environment. For example, Radhakrishnan *et al*. [4] found 92% of 200 interviewed mahouts in India had been attacked at least once by their elephants (45% resulting in serious injuries), and Lair [2] estimated Burmese mahouts to have a 1/5 chance of being killed by an elephant in their lifetime based on a reported 20 mahout deaths/year, while there have been reports of 200 mahout deaths/year in Thailand [55]. Therefore, future recommendations must prioritize mahout safety as well as elephant welfare while respecting the knowledge and expertise of mahouts. Bansiddhi *et al*. [29], for example, praised McLean for preserving mahout customs by incorporating the ankus into positive training techniques [56,57].

In conclusion, physiological stress indicators increased during taming, suggesting we should also monitor calf health during this time as heightened, prolonged physiological stress is often linked to pathological issues. It would also be beneficial for future studies to focus on long-term impacts, for example, through biological ageing markers to understand how taming might affect individuals’ allostatic load and long-term fitness, as well as monitoring their behaviour as a complementary welfare indicator. It could also be informative to monitor a full diurnal profile of HPA activity to explore cortisol changes throughout the day [25]. Studies should extend to populations employing different training methods to compare both the physiological impact of different methods and behavioural outcomes relevant to mahout safety and elephant working ability in free contact. Traditional elephant taming is often a taboo subject among welfare specialists, yet it is linked to the livelihoods and well-being of thousands of mahouts. As stated by Laule & Whittaker [58] about protected contact, a similarly sensitive topic in elephant management, we must ‘move past differences in opinion into the realm of fact and objective assessment’ to make evidence-based decisions optimizing conditions for elephants and mahouts [59]. Furthermore, while aspects of taming in this population may compromise calf welfare reflected in their heightened physiological stress indicators, there are probably management factors reducing stress, such as the constant supply of natural fodder and water, veterinary care and prolonged time with their mother, which are missing in other populations [5]. Comparisons to other populations will allow us to identify these important factors with the aim to improve the welfare of thousands of Asian elephants that live in captivity around the world.

## Data Availability

All data to perform analyses and recreate figures can be found in the electronic supplementary material [60]. A 'Readme.txt' file details all 14 supplementary files (one pdf, 2 R scripts and 11 csv files).
